# Pro-inflammatory activity of long noncoding RNA FOXD2-AS1 in Achilles tendinopathy

**DOI:** 10.1186/s13018-023-03681-0

**Published:** 2023-05-16

**Authors:** Xiaoting Ke, Wenjie Zhang

**Affiliations:** 1 Zhejiang Rehabilitation Medical Center, Hangzhou, 310051 Zhejiang China; 2 Center for Rehabilitation Medicine, Rehabilitation & Sports Medicine Research Institute of Zhejiang Province, Department of Rehabilitation Medicine, Zhejiang Provincial People’s Hospital (Affiliated People’s Hospital, Hangzhou Medical College), Hangzhou, 310014 Zhejiang China

**Keywords:** Long noncoding RNA FOXD2-AS1, microRNA-21-3p, PTEN, PI3K/AKT signaling pathway, Achilles tendinopathy, Inflammatory response

## Abstract

Achilles tendinopathy is a prevalent clinical problem that plagues athletes and general populations. Achilles tendon healing is a complex process, and so far, there is no successful long-term solution to Achilles tendinopathy in the field of microsurgery due to its poor natural regeneration ability. Limitations in understanding the pathogenesis of Achilles tendon development and Achilles tendon injury hinder clinical treatment developments. There is an increasing demand for innovative conservative treatments that can improve Achilles tendon injury. In this study, a Sprague–Dawley rat model of Achilles tendinopathy was established. Lentiviral vectors that interfere with the expression of FOXD2-AS1, miR-21-3p, or PTEN were injected every 3 days. Rats were euthanized after 3 weeks, and the effect of FOXD2-AS1, miR-21-3p, or PTEN on Achilles tendon healing was analyzed by histological observation, biomechanical test, and examinations of inflammatory factors and tendon markers. As measured, downregulating FOXD2-AS1 or upregulating miR-21-3p improved histological structure, suppressed inflammation, promoted the expression of tendon markers, and optimized the biomechanical properties of Achilles tendon. Upregulating PTEN was capable of reversing the promoting effect of inhibition of FOXD2-AS1 on Achilles tendon healing. As concluded, deficiency of FOXD2-AS1 accelerates the healing of Achilles tendon injury and improves tendon degeneration by regulating the miR-21-3p/PTEN axis and promoting the activation of the PI3K/AKT signaling pathway.

**Supplementary Information:**

The online version contains supplementary material available at 10.1186/s13018-023-03681-0.

## Introduction

Achilles tendinopathy (AT) is a common cause of disability in many athletes due to the continuous prolonged intense functional demands imposed on the Achilles tendon [1]. Symptomatic AT often causes localized pain, swelling, and dysfunction around tendons [2–4], which can seriously affect people's daily life or athletes’ careers. However, the cause of AT is currently unknown. Therefore, symptomatic treatment, such as analgesics, anti-inflammatory drugs, and functional rehabilitation training, is usually used clinically, and surgical treatment is only performed when Achilles tendon is injured or ruptured [4]. There are many studies on the pathogenesis of AT, including inflammation, micro-injury, oxidative stress, degeneration, etc. [5–8]. Overload and use of the Achilles tendon can cause local inflammation or microdamage, which leads to changes in the extracellular microenvironment and matrix metalloproteinases production. This triggers the rebuilding of collagen fibers (the main component changes from type I collagen to type III collagen), the formation of microvessels, and the degeneration of a large number of tendon cells, thereby leading to AT [9]. The tendon healing process is often slow and incomplete, with an augmented incidence of degenerative events associated with a poor response to treatments [10]. Much attention is currently paid to the development of innovative and effective conservative approaches suitable as adjuvants to surgical intervention to promote tissue healing [11].

Long noncoding RNAs (lncRNAs) are in essence in transcriptional and post-transcriptional regulation of gene expression and are the regulatory center of target gene transcriptional activity and mRNA expression [12]. Accumulating evidence suggests that lncRNAs are closely associated with the progression of a variety of diseases [13–16]. However, the exact functions and underlying mechanisms of lncRNAs in Achilles tendinopathy remain unclear. A study notes that lncRNA FOXD2-AS1 (FOXD2-AS1) induces chondrocyte proliferation, inflammation, and extracellular matrix degradation in osteoarthritis [17].

MicroRNAs (miRNAs) can inhibit gene expression by binding to the 3'UTR of target mRNAs [18]. miRNAs are important regulators of cell proliferation, differentiation, inflammatory response, and apoptosis [19]. In recent years, more and more studies have shown that miRNAs play an important regulatory role in musculoskeletal diseases [20, 21]. It has been reported that administration of double-stranded miR-210 in a rat model promotes early Achilles tendon healing [22], and miR-29b mediates fibroblast growth in Achilles tendon [23]. miR-21-3p is a comprehensive miRNA associated with the progression of various diseases. It has been reported that miR-21-3p can regulate inflammatory pathways [24], and modification of miR-21-3p is of significance in inhibiting tendon adhesion [25].

To this end, this study constructed a rat Achilles tendinopathy model to assess tissue morphological changes, inflammation, collagen fiber remodeling, and Achilles tendon healing to determine the pathophysiological relationship between FOXD2-AS1, miR-21-3p, and Achilles tendinopathy. Our findings will provide a therapeutic exploration of Achilles tendinopathy and a better understanding of the regulatory roles of FOXD2-AS1 and miR-21-3p in this process.

## Materials and methods

### Establishment of a rat model of Achilles tendinopathy

The animal experimental protocol was approved by the Animal Research Committee of Zhejiang Provincial People’s Hospital. Forty-eight 8-week-old male SD rats, weighing about 200–250 g (JKbiot, Nanjing, China), were housed in ventilated micro-isolated cages with a 12-h light and dark cycle. Rats were anesthetized with 2.5% pentobarbital sodium (0.25 ml/100 g) and injected with bacterial collagenase I (20 μl 0.015 mg/ml saline; Sigma-Aldrich) or saline into the Achilles tendon with a 29-gauge needle on one side [26]. After 3 days, lentiviruses (1 × 10^9^ TU/ml) or negative control lentiviruses (GenePharma) interfering with FOXD2-AS1, miR-21-3p, or PTEN expression or negative control were injected into the Achilles tendon. After 3 weeks, the rats were euthanized, and Achilles tendon tissue was collected for histological and biomechanical analysis.

To generate lentiviruses that interfere with FOXD2-AS1, miR-21-3p, or PTEN expression, si-FOXD2-AS1, miR-21-3p agomir, or oe-PTEN was subcloned into the lentivirus vector pLV-CMV. The construct was then transfected into 293 T cells with auxiliary vectors pSPAX2 and pMD2G to generate lentivirus (1 × 10^9^ TU/ml).

### Histological observations

The Achilles tendon tissue was prepared into paraffin tissue Sects. (4 μm) for hematoxylin and eosin (H&E) staining. Immunohistochemical staining of Achilles tendon tissue was performed using primary antibodies COL1A1 (1:300, Abcam, ab34710) and TNMD (1:100, Abcam, ab203676) and the secondary antibody (1:200, Cell Signaling Technology) [27]. Positive staining was semi-quantitatively analyzed by Image J software.

### Biomechanical tests

The specimen was carefully mounted on the material testing system (TytronTM 250, MTS Systems Corporation, Eden Prairie, USA) to prevent twists in the tendon using a custom-designed clamp. The specimen was immersed in a PBS bath and the temperature was controlled at 25 °C via a temperature regulator throughout the overall biomechanical testing to keep the specimen moist and at a constant temperature, and to provide the medium needed for the US examination (Additional file 1: Figure S1). Tensile tests were performed (axial velocity: 30 mm/min, 0.1-N preload using a 100-N force transducer) until failure at maximum load. The biomechanical properties of the Achilles tendon were evaluated by ultimate tensile strength (UTS) (N), stiffness (N/mm), and Young's modulus [26].

### ELISA

The Achilles tendon tissue was homogenized with a lysis buffer containing protease inhibitors and centrifuged at 14,000 g for 25 min. Interleukin-1β (IL-1β) and tumor necrosis factor (TNF-α) levels in the supernatant were detected by ELISA kits (Invitrogen, CA, USA).

### RNA expression quantification

Total RNA was extracted using the Rneasy Mini Kit (Qiagen, Valencia, CA, USA) and reverse transcribed to cDNA using the PrimeScript RT Kit (Takara, Dalian, China) or the miScript II Reverse Transcriptase Kit (Qiagen). Then, qPCR was performed using BeyoFast SYBR Green qPCR Mix (Beyotime, Shanghai, China). Expression was examined using the 2^−ΔΔCt^ method with glyceraldehyde-3-phosphate dehydrogenase (GAPDH) or U6 as internal controls. The primer sequences are shown in Table 1.

**Table 1:** PCR primers

Genes	Primers (5′–3′)
FOXD2-AS1	Forward: GCCCAGAACAATTGGGAGGA
	Reverse: AAGAGAGGGAGAGACGACCC
miR-21-3p	Forward: CAACAGCAGTCGATGGG
	Reverse: GCAGGGTCCGAGGTATTC
PTEN	Forward: AGAGGAGCCGTCAAATCCAG
	Reverse: TCTCTGGATCAGAGTCAGTGGT
U6	Forward: CTCGCTTCGGCAGCACA
	Reverse: AACGCTTCACGAATTTGCGT
GAPDH	Forward: GTCGGTGTGAACGGATTTG
	Reverse: TCCCATTCTCAGCCTTGAC

FOXD2-AS1, Long noncoding RNA FOXD2 adjacent opposite strand RNA1; miR-21-3p, microRNA-21-3p; PTEN, phosphatase and tensin homolog; GAPDH, glyceraldehyde-3-phosphate dehydrogenase

### Immunoblotting

Total protein was collected by lysing tissues in the radioimmunoprecipitation buffer and quantified by BCA kit. Total proteins were electrophoretically transferred to PVDF membranes followed by sequential incubation with primary antibodies PTEN (SC-7974, 1:500, Santa Cruz Biotechnology), p-PI3K (4228, 1:1000, Cell Signaling Technology), p-AKT (9271, 1:1000, Cell Signaling Technology), and GAPDH (ab8245, 1:1000, Abcam) and HRP-conjugated secondary antibody (1:5000, Cell Signaling Technology). An enhanced chemiluminescence solution (GE LifeScience) and an imaging system (Bio-Rad, CA, USA) were utilized for band visualization and Image J software was for data quantification.

### Dual-luciferase reporter gene assay

The luciferase reporters (FOXD2-AS1-WT, FOXD2-AS1-MUT, PTEN 3’UTR-WT, and PTEN 3’UTR-MUT) were established by inserting the 3'UTR sequences of FOXD2-AS1 and PTEN containing the binding site of wild-type or mutant miR-21-3p into pmirGLO vector (Promega). The reporters and miR-21-3p mimic or mimic NC were co-transfected into 293 T cells using Lipofectamine 2000 (Invitrogen). The luciferase activity was detected at 48 h using a dual-luciferase reporter gene assay kit (Promega).

### Statistical analysis

Data were presented as mean ± standard deviation. Statistical significance was determined using Tukey’s multiple comparison test and two-way ANOVA. *P* value less than 0.05 was considered significant. Statistical analysis of all graphs was performed using GraphPad prism 8.2.1.441.

## Results

### Manifestation and effects of FOXD2-AS1 in a rat model of Achilles tendinopathy

**Fig. 1:**
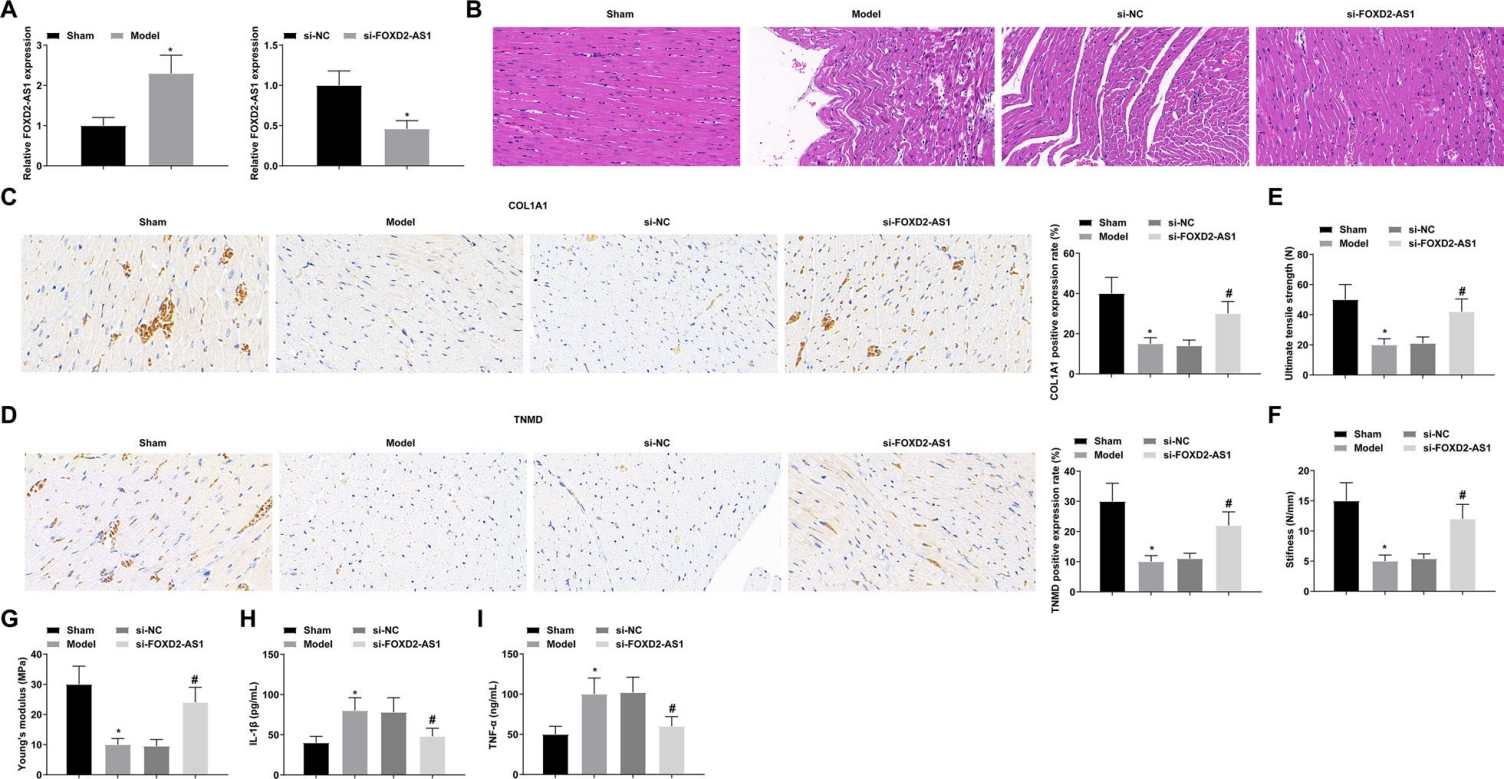
Manifestation and effects of FOXD2-AS1 in a rat model of Achilles tendinopathy. **A** RNA expression quantification of FOXD2-AS1; **B** HE staining of Achilles tendon; **C**–**D** Immunohistochemical positive staining of COL1A1 and TNMD; **E**–**G** UTS, stiffness, and Young's modulus; **H**–**I** ELISA analysis of IL-1β and TNF-α in rat Achilles tendon; the values were expressed as mean ± standard deviation. *P < 0.05 versus Sham; # *P* < 0.05 versus si-NC

Based on a collagenase I-induced Achilles tendinopathy model, si-FOXD2-AS1 lentivirus was injected. FOXD2-AS1 expression in Achilles tendon tissue was quantitatively analyzed, presenting an upward trend in rats with Achilles tendinopathy. After injection with si-FOXD2-AS1 lentivirus, FOXD2-AS1 expression was decreased (Fig. 1A). Histological observations by HE staining and immunohistochemistry indicated that after downregulating FOXD2-AS1, the degenerative changes of Achilles tendon were alleviated, and the level of collagen fiber rupture was reduced (Fig. 1B), and the positive expression of tendon markers COL1A1 and TNMD was elevated (Fig. 1C, D). Meanwhile, biomechanical tests indicated that UTS, stiffness, and Young's modulus of the Achilles tendon were enhanced after downregulating FOXD2-AS1 (Fig. 1E–G). Additionally, ELISA detection of rat Achilles tendon tissue found that suppression of FOXD2-AS1 alleviated the inflammatory response by reducing IL-1β and TNF-α contents (Fig. 1H, I). Taken together, FOXD2-AS1 inhibition promotes injury healing, improves tendon degeneration, enhances biomechanical properties, and reduces inflammation in Achilles tendon.

### Interplay between FOXD2-AS1 and miR-21-3p

**Fig. 2:**
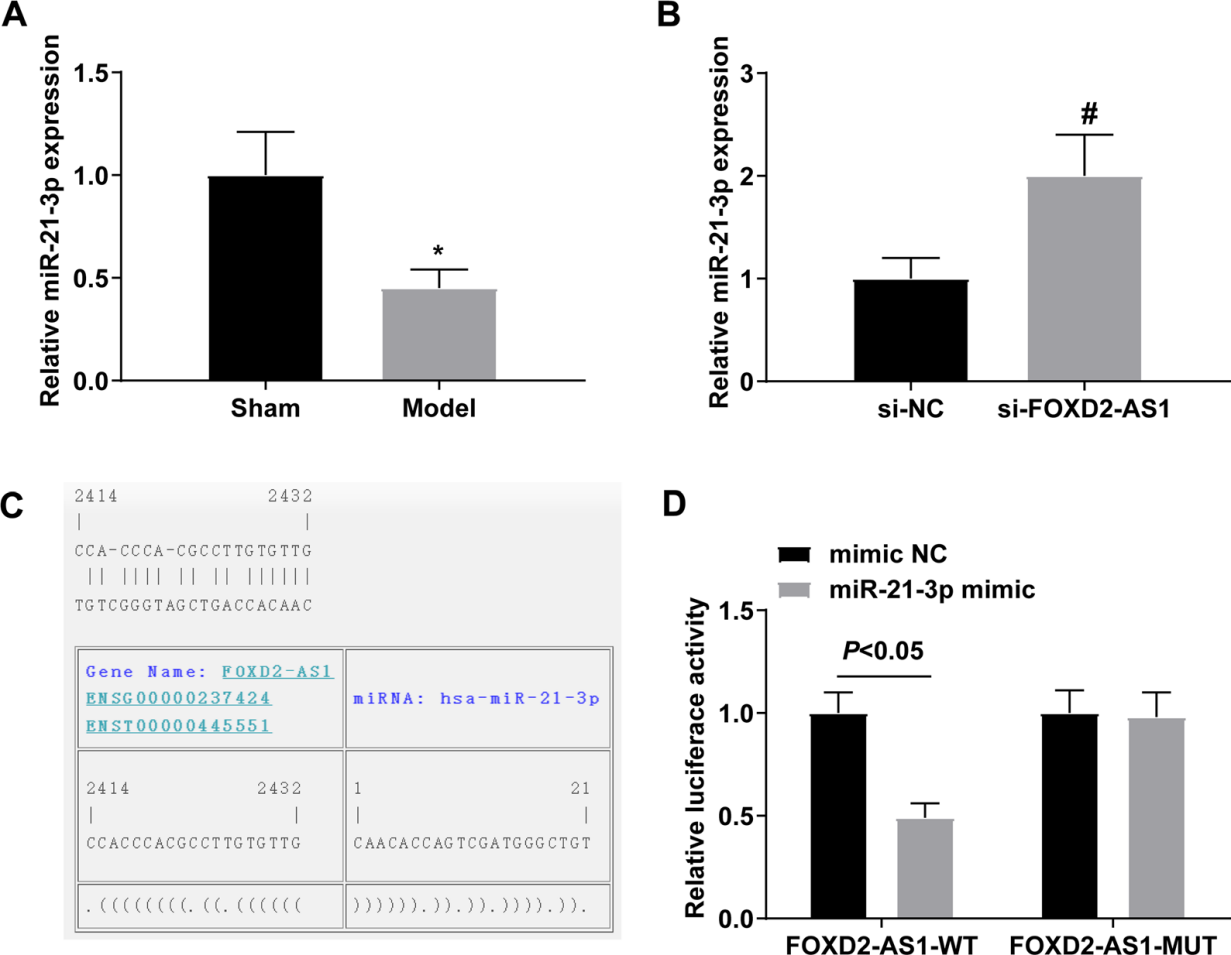
Interplay between FOXD2-AS1 and miR-21-3p. **A** RNA expression quantification of miR-21-3p in Achilles tendinopathy rats; **B** RNA expression quantification of miR-21-3p after downregulating FOXD2-AS1; **C** The binding site of FOXD2-AS1 and miR-21-3p on starBase; D. Verification of the targeting relationship between FOXD2-AS1 and miR-21-3p; values were expressed as mean ± standard deviation. *P < 0.05 versus Sham; # *P* < 0.05 versus si-NC

miR-21-3p was found to be downregulated in Achilles tendinopathy rats (Fig. 2A) but elevated in FOXD2-AS1-deficient Achilles tendinopathy rats (Fig. 2B). Based on these results, there might be a targeting relationship between FOXD2-AS1 and miR-21-3p. starBase predicted that FOXD2-AS1 shared a binding site with miR-21-3p (Fig. 2C) and dual-luciferase experiments confirmed that co-transfection of FOXD2-AS1-WT with miR-21-3p mimic reduced cellular luciferase activity (Fig. 2D). Collectively, FOXD2-AS1 interacts with miR-21-3p.

### Therapeutic outcomes of miR-21-3p in a rat model of Achilles tendinopathy

**Fig. 3:**
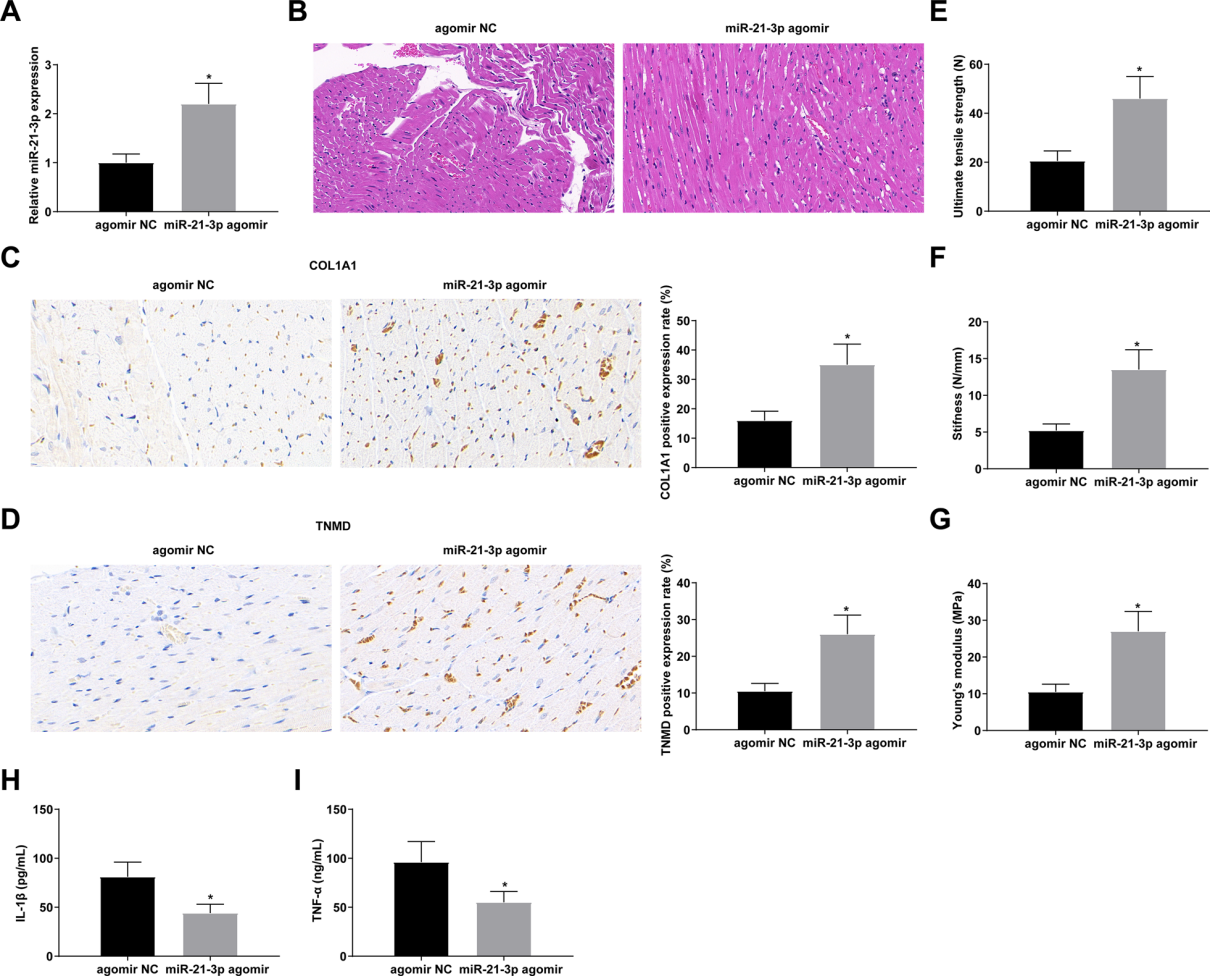
Therapeutic outcomes of miR-21-3p in a rat model of Achilles tendinopathy. **A** RNA expression quantification of miR-21-3p; **B** HE staining of Achilles tendon; **C**–**D** Immunohistochemical positive staining of COL1A1 and TNMD; **E**–**G** UTS, stiffness, and Young’s modulus; **H**–**I** ELISA analysis of IL-1β and TNF-α in rat Achilles tendon; values were expressed as mean ± standard deviation. *P < 0.05 versus agomir NC

Based on the Achilles tendinopathy model, miR-21-3p agomir lentivirus was injected, resulting in the increase in miR-21-3p expression (Fig. 3A). Benefiting from miR-21-3p upregulation, rat Achilles tendon degeneration was alleviated, collagen fiber rupture was reduced (Fig. 3B), COL1A1 and TNMD expression was elevated (Fig. 3C, D), UTS, stiffness, and Young's modulus were enhanced (Fig. 3E–G), and inflammatory response was limited (Fig. 3H, I). In conclusion, miR-21-3p accelerates the healing of Achilles tendon injury and improves tendon degeneration.

### Regulation of PTEN by miR-21-3p

**Fig. 4:**
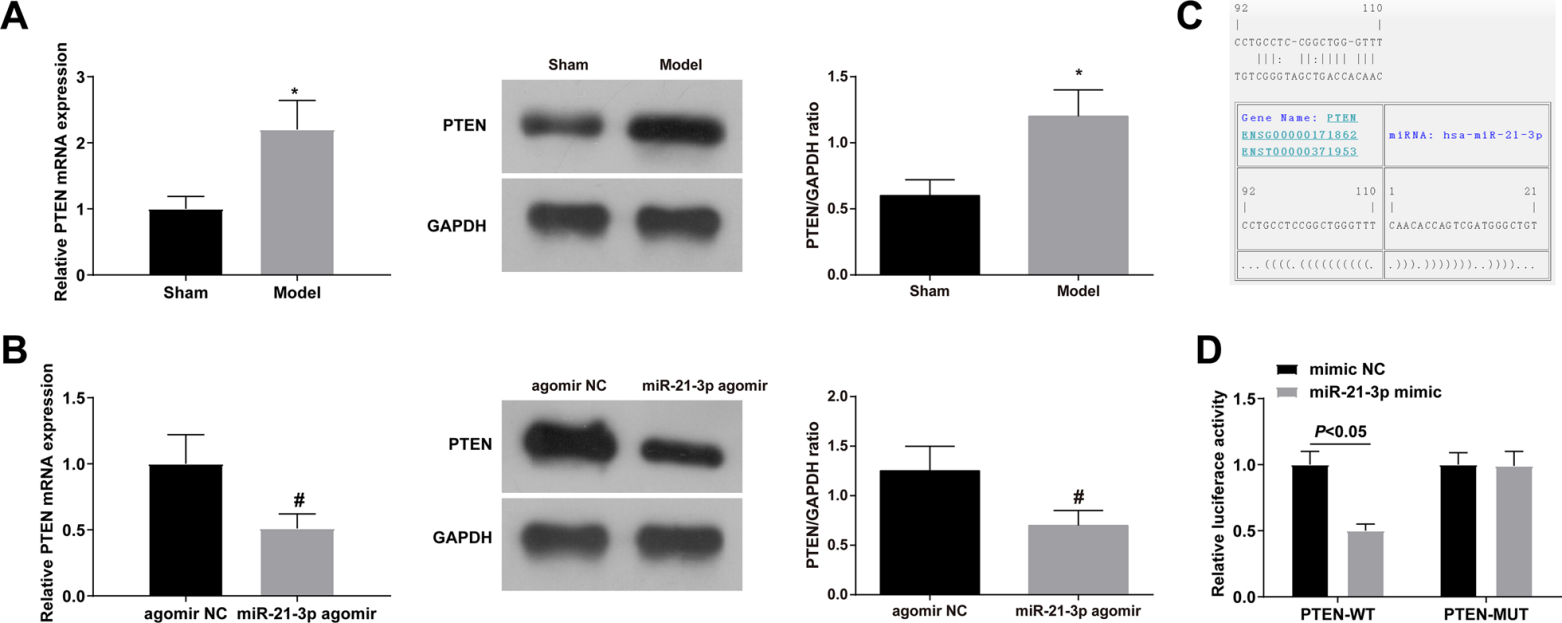
Regulation of PTEN by miR-21-3p. **A** RNA expression quantification and immunoblot of PTEN in Achilles tendinopathy rats; **B** RNA expression quantification and immunoblot of PTEN after upregulating miR-21-3p; **C** Binding site of miR-21-3p and PTEN on starBase database; **D** Verification of the targeting relationship between miR-21-3p and PTEN; values were expressed as mean ± standard deviation. *P < 0.05 versus Sham; #*P* < 0.05 versus agomir NC

An increase was observed in PTEN expression in rats with Achilles tendinopathy (Fig. 4A) while a decrease was noticed after elevating miR-21-3p (Fig. 4B). miR-21-3p had a binding site with PTEN through the starBase (Fig. 4C) and this binding relation was validated by luciferase activity measurements (Fig. 4D). To sum up, miR-21-3p targets the regulation of PTEN expression.

### PTEN can reverse the promoting effect of down-regulation of FOXD2-AS1 on Achilles tendon healing

**Fig. 5:**
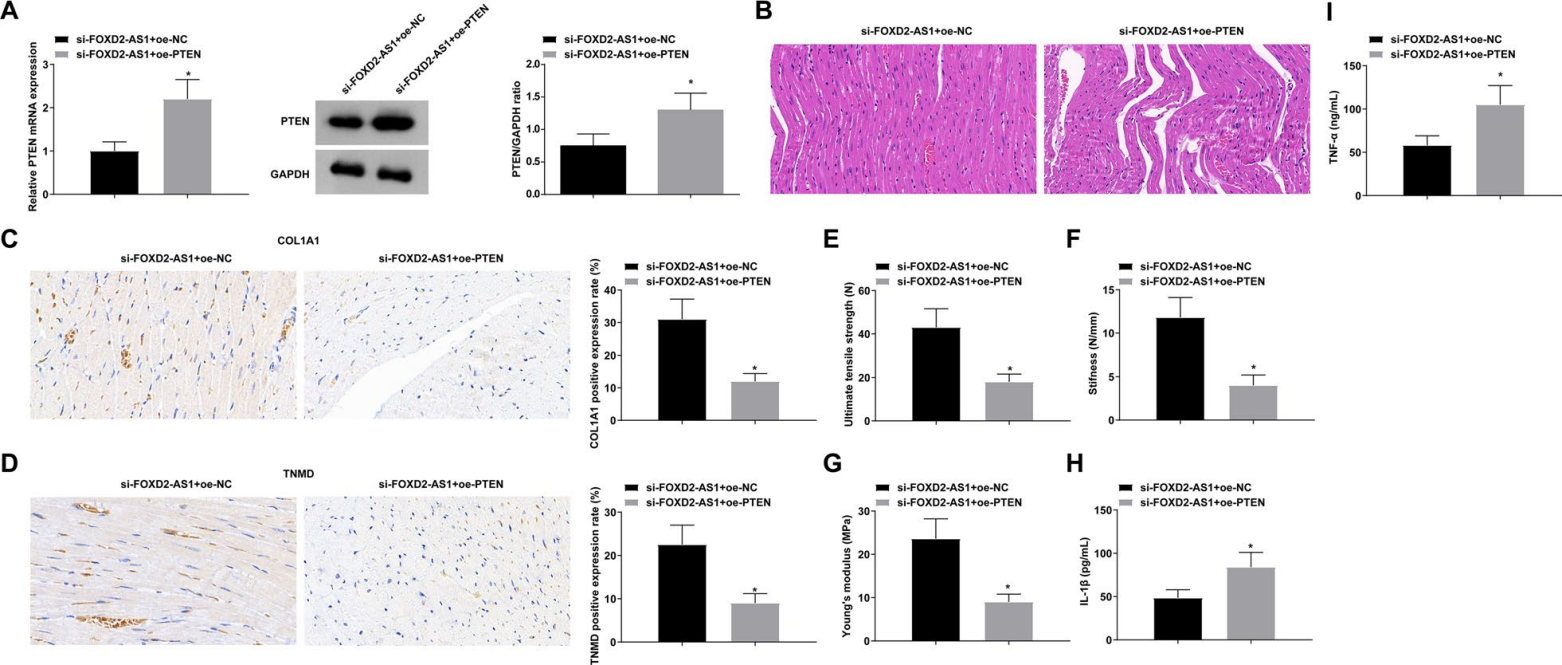
PTEN can reverse the promoting effect of down-regulation of FOXD2-AS1 on Achilles tendon healing. **A** RNA expression quantification and immunoblot of PTEN; **B** HE staining of Achilles tendon; **C**–**D** Immunohistochemical positive staining of COL1A1 and TNMD; **E**–**G** UTS, stiffness, and Young’s modulus; **H**–**I** ELISA analysis of IL-1β and TNF-α in rat Achilles tendon; values were expressed as mean ± standard deviation. *P < 0.05 versus si-FOXD2-AS1 + oe-NC

Si-FOXD2-AS1 and oe-PTEN lentiviruses were injected in rats with Achilles tendinopathy. The successful injection was verified as evidenced by the upregulation of PTEN (Fig. 5A). Various experiments revealed that induction of PTEN abolished the promoting effect of down-regulation of FOXD2-AS1 on Achilles tendon healing (Fig. 5B–I).

### FOXD2-AS1 inhibits the PI3K/AKT signaling pathway activation by regulating miR-21-3p/PTEN axis

**Fig. 6:**
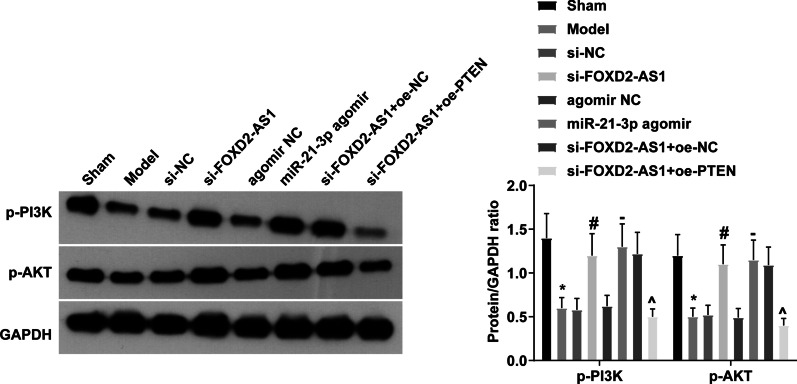
FOXD2-AS1 inhibits PI3K/AKT signaling pathway activation by regulating miR-21-3p/PTEN axis. Immunoblot of p-PI3K and p-AKT; the values are expressed as mean ± standard deviation; *P < 0.05 versus Sham; # *P* < 0.05 versus si-NC;—*P* < 0.05 versus agomir NC; ^*P* < 0.05 versus si-FOXD2-AS1 + oe-NC

Targeted control of the PI3K/AKT pathway can treat Achilles tendinitis [28]. PI3K/AKT signaling pathway-related factors were analyzed, and p-PI3K and p-AKT protein expression was decreased in rats with Achilles tendinopathy while these changes were reversed after downregulating FOXD2-AS1 or upregulating miR-21-3p; upregulating PTEN could reverse the promotion of p-PI3K and p-AKT expression mediated by down-regulation of FOXD2 (Fig. 6). Collectively, FOXD2-AS1 inhibits the activation of PI3K/AKT signaling pathway by regulating the miR-21-3p/PTEN axis.

## Discussion

In recent years, there has been increasing interest in noncoding RNAs-mediated epigenetic regulation of transcription in diverse biological functions [29, 30]. The function of miRNAs in Achilles tendinopathy has been investigated, but the role of lncRNAs remains largely obscure. FOXD2-AS1 was originally identified as a tumor promoter in various human cancers [31–33]. In recent years, FOXD2-AS1 has been revealed to be involved in chondrocyte proliferation, inflammation, and extracellular matrix degradation in osteoarthritis [17, 34]. Based on this, this study mainly explored the role and possible molecular mechanisms of FOXD2-AS1 in Achilles tendinopathy.

Animal models are often utilized to study the pathogenesis of Achilles tendinopathy [35]. Type I collagen is enriched in tendon matrix [36] and is the primary collagen that binds to the tendon structure, and increased production of type I collagen promotes tendon healing, whereas destruction of type I collagen is detrimental to tendon healing [37, 38]. Therefore, this study established a rat Achilles tendinopathy model by injecting collagenase I into the Achilles tendon, and injected si-FOXD2-AS1 lentivirus into rats to explore the role of FOXD2-AS1 in Achilles tendinopathy. This study found that after downregulating FOXD2-AS1, the degenerative changes of rat Achilles tendon were alleviated, collagen fiber rupture was reduced, COL1A1 and TNMD expression was increased, and UTS, stiffness, and Young’s modulus of Achilles tendon were increased. Several studies have shown that inflammation is closely related to tendon healing [2, 39–42], and chronic excessive inflammation promotes tendon degeneration and affects tendon repair and reconstruction [43, 44]. TNF-α and IL-6 are typical pro-inflammatory cytokines [45]. This study detected the reduced contents of IL-1β and TNF-α in rat Achilles tendon tissue after downregulating FOXD2-AS1.

Mechanistically, as previously described [46], a major mechanism by which lncRNAs regulate miRNA expression and activity as ceRNAs. In addition, recent studies have elucidated the role of miRNAs in the repair of Achilles tendon injury [22, 23]. Against this background, this study was interested in the downstream miRNAs of FOXD2-AS1 and finally confirmed that miR-21-3p was the target gene of FOXD2-AS1. miR-21-3p is involved in improving tendon adhesion [25]. This study confirmed that miR-21-3p was downregulated in Achilles tendinopathy rats and upregulating miR-21-3p could improve Achilles tendon histopathology, inhibit inflammation, and optimize the biomechanical properties of Achilles tendon in rats with Achilles tendinopathy.

This research set out to define the downstream potential mechanisms of miR-21-3p and noticed a binding relation between PTEN and miR-21-3p. In addition, this study also confirmed that PTEN expression was elevated in rats with Achilles tendinopathy. PTEN is a prominent tumor suppressor gene [47] that inhibits the PI3K/AKT pathway through lipid phosphatase activity [48, 49]. Recently, PTEN has been revealed to be involved in tendon healing and regeneration [50]. Based on this, we further explored the regulatory role of the FOXD2-AS1/miR-21-3p/PTEN axis in rats with Achilles tendinopathy. The experimental results indicated that upregulating PTEN could reverse the promoting effect of down-regulation of FOXD2-AS1 on Achilles tendon healing.

PTEN/PI3K/AKT signaling pathway modifies various functions of cells [51] and the PI3K/AKT pathway can be a target signaling to treat Achilles tendinitis [28]. Furthermore, it has been reported that the PTEN/PI3K/AKT axis is involved in embryonic bone development and fracture healing [52]. More importantly, miR-21-3p knockdown can inhibit the PI3K/AKT signaling pathway by targeting PTEN [53] and FOXD2-AS1 activates PI3K/AKT signaling pathway [54]. Based on this, the present study further verified that PI3K/AKT signaling pathway was activated in Achilles tendinopathy rats, inhibition of FOXD2-AS1 or induction of miR-21-3p could activate the PI3K/AKT signaling pathway, while overexpression of PTEN mitigated the impact of silenced FOXD2 on PI3K/AKT signaling pathway. All in all, our findings suggest that FOXD2-AS1 alleviates the progression of Achilles tendinopathy and the underlying mechanism is mediated, at least in part, through the PI3K/AKT signaling pathway targeting PTEN through miR-21-3p.

However, our findings are subject to certain limitations. For example, our findings are based on in vivo animal experiments, and in vitro cellular experiments have not been investigated. Furthermore, activation of the PI3K/AKT signaling pathway mediates the progression of Achilles tendinopathy remains obscure.

## Conclusion

Our results suggest that down-regulation of FOXD2-AS1 can accelerate the healing of Achilles tendon injury and improve Achilles tendon degeneration by regulating the miR-21-3p/PTEN axis and promoting the activation of the PI3K/AKT signaling pathway. These findings may provide new ideas and targets for the treatment of Achilles tendinopathy.

## Supplementary Information

**Additional file 1**.** Figure S1**: Experimental apparatus for measuring biomechanic.

## Data Availability

Data are available from the corresponding author on request.
