# Supplementary figures and images for: Pro-inflammatory activity of long noncoding RNA FOXD2-AS1 in Achilles tendinopathy
Source: J Orthop Surg Res. 2023 May 16;18:361. doi: 10.1186/s13018-023-03681-0 (PMC10189999; doi:10.1186/s13018-023-03681-0)

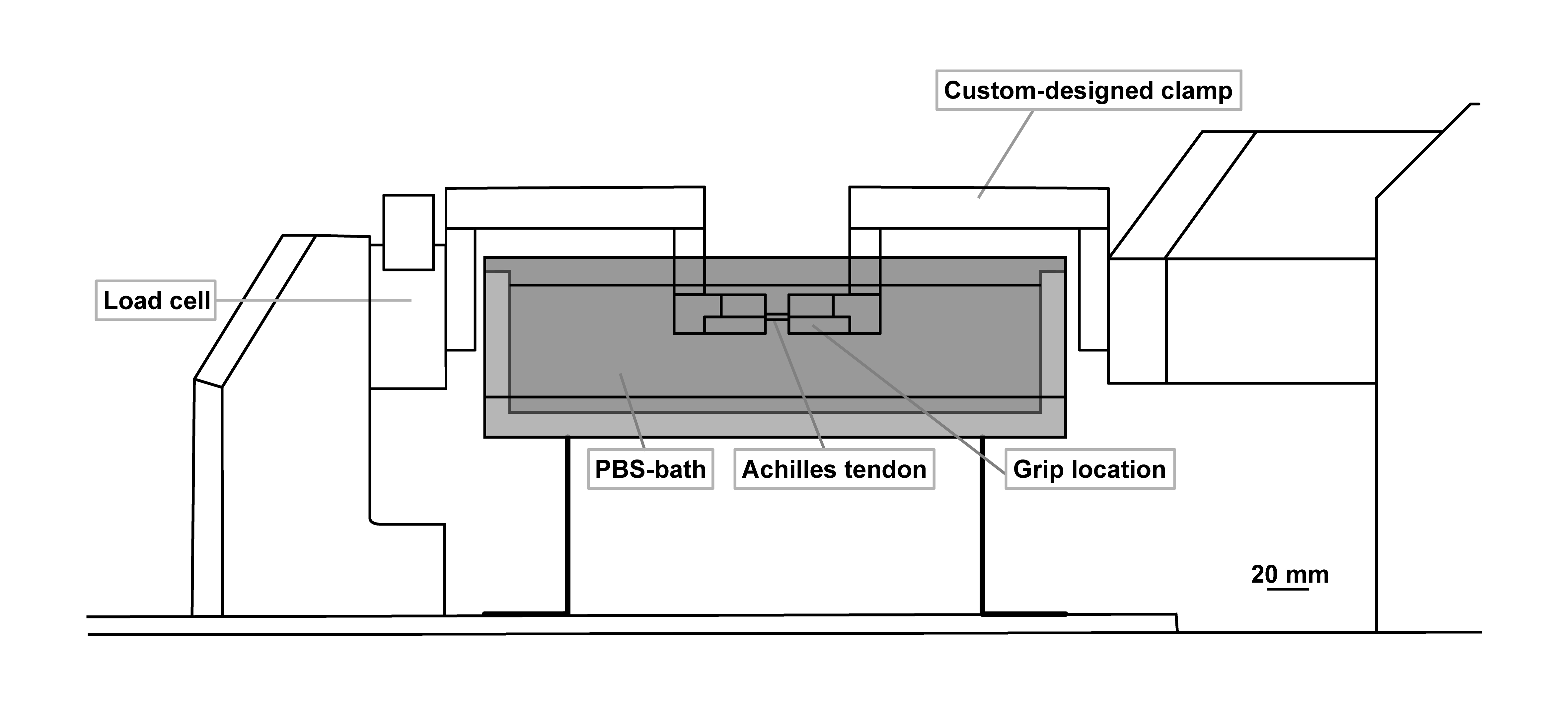

Supplement: Supplementary file 1 — Additional file 1. Figure S1: Experimental apparatus for measuring biomechanic. [file 13018_2023_3681_MOESM1_ESM.tif]
